# Longitudinal measurement invariance of the patient health questionnaire in a German sample

**DOI:** 10.1186/s12888-021-03390-0

**Published:** 2021-08-04

**Authors:** Anne Moehring, Diana Guertler, Kristian Krause, Gallus Bischof, Hans-Juergen Rumpf, Anil Batra, Susanne Wurm, Ulrich John, Christian Meyer

**Affiliations:** 1 Department of Social Medicine and Prevention, Institute of Community Medicine, University Medicine Greifswald, Walther-Rathenau-Str. 48, 17475 Greifswald, Germany; 2 DZHK (German Center for Cardiovascular Research), Partner Site Greifswald, Greifswald, Germany; 3 Institute for Medical Psychology, University Medicine Greifswald, Walther-Rathenau-Str. 48, 17475 Greifswald, Germany; 4 Department of Psychiatry and Psychotherapy, Research Group S:TEP, University of Luebeck, Ratzeburger Allee 160, 23538 Luebeck, Germany; 5 Department of Psychiatry and Psychotherapy, University Hospital of Tuebingen, Calwer Str. 14, 72076 Tuebingen, Germany

**Keywords:** Measurement invariance, Patient health questionnaire, PHQ-8, CFA, Factor structure

## Abstract

**Background:**

The Patient Health Questionnaire-8 (PHQ-8) is a screening questionnaire of depressive symptoms. However, it is unknown whether it is equivalent across time and between groups of individuals. The aim of our paper was to test whether the PHQ-8 has the same meaning in two groups of individuals over time.

**Methods:**

Primary care patients were proactively recruited from three German cities. PHQ-8 data from a baseline assessment (*n* = 588), two assessments during the intervention (*n* = 246/225), and a six (*n* = 437) and 12 months (*n* = 447) follow-up assessment were first used to examine the factor structure of the PHQ-8 by confirmatory factor analysis (CFA). The best fitting factor solution was then used to test longitudinal invariance across time and between intervention and control group by Multiple Group CFA.

**Results:**

A two-factor structure consistently showed the best model fit. Only configural longitudinal invariance was evidenced when the baseline assessment was included in the analysis. Without the baseline assessment, strict longitudinal invariance was shown across the intervention and the follow-up assessments. Scalar invariance was established between the intervention and control group for the baseline assessment and strict invariance between groups and across the 6- and 12-month follow-up assessments.

**Conclusions:**

The lack of longitudinal invariance might be attributed to various differences between the baseline assessments and all following assessments, e.g., assessment mode (iPad vs telephone), potential changes in symptom perception, and setting.

**Trial registration:**

DRKS00011635, date of trial registration: 20.01.2017; DRKS00011637, date of trial registration: 25.01.2017.

**Supplementary Information:**

The online version contains supplementary material available at 10.1186/s12888-021-03390-0.

## Background

Depression is one of the most prevalent and burdensome mental health disorders worldwide. The World Health Organization (WHO) calls it one of the top risk factors for health and predicts depression and affective disorders will be the second most frequent widespread disease worldwide by 2020 [1]. Standardized clinical interviews such as the Composite International Diagnostic Interview (CIDI, [2]) are valid and reliable instruments to assess depression [3–6]. However, their administration is time-consuming and requires trained interviewers. Therefore, shorter self-report measures are often used instead of clinical interviews in population-based surveys to screen for depression. The Patient Health Questionnaire-9 (PHQ-9, [7]) is a nine-item self-report measure of depressive symptoms that has been used in clinical and general population samples [8–10]. The questionnaire has been translated into several languages for widespread international use (e.g., [11–13]). The nine items represent the nine clinical criteria for depression from the Diagnostic and Statistical Manual of Mental Disorders, fifth edition (DSM-5, [14]): anhedonia, depressed mood, sleep disturbance, fatigue, appetite changes, low-self-esteem, concentration problems, psychomotor disturbances, and suicidal ideation. Thus, the PHQ-9 screens for affective, cognitive, and somatic aspects of depression. In intervention studies, the PHQ-9 is frequently used as a measure of changes in depression severity [15–17]. The PHQ-9 has been validated as self-administered questionnaire [7, 11] and as telephone interview [18]. It may be used in clinical and non-clinical samples [10]. Another widely used version of this questionnaire is the PHQ-8 [19]. It is a short version of the PHQ-9, which has one additional item on self-injurious or suicidal ideas. However, data revealed that this item was often superfluous for assessments because thoughts of self-harm are rather uncommon even in samples of clinically depressed patients [20, 21]. Furthermore, some studies suggest that this item shows a notably low discriminatory power [8] and often indicates passive thoughts about death rather than suicidal or self-harm intentions [22]. This confirms the suitability of the PHQ-8, which has shown good validity and reliability as a measure of different levels of depression. Still, most research on psychometric properties has been done using the PHQ-9.

Research has been undertaken to assess whether the PHQ-9 includes different subscales that indicate different symptom domains. For this purpose, its psychometric factor structure has been analyzed. Several findings on the factor structure of the PHQ-9 exist. They provide support for a one-factor [23–26], a two-factor model [27–29] or, albeit less frequently, a three-factor model [30]. Overall, the results regarding the factor structure are still inconsistent. In their systematic review, Lamela, Soreira [29] provide an overview of the heterogeneity in the factor structure of the PHQ-9. Their own results support the two-factor structure of the questionnaire. Similarly, Mattsson, Sandqvist [31] found a two-factor structure for the PHQ-8. A two-factor structure was also found in a sample of patients with chronic heart failure [32]. However, using exploratory factor analysis, Schantz, Reighard [33] found a one-factor structure of the PHQ-8.

Measurement invariance is a crucial prerequisite for comparisons between groups of individuals and points of time in measurement. If measurement invariance is evidenced, we can conclude that the same construct is measured across groups and that observed group differences reflect true group differences. Failure to obtain measurement invariance renders group comparisons ambiguous because they might merely be caused by psychometric differences related to item responses instead of differences in the underlying construct. There are studies on the measurement invariance of the PHQ-9, especially in regards to gender specific measurement invariance [9, 34]. However, there is a need for the comparison of groups for studies with experimental designs. In order to assume that we interpret true group differences when examining differences between intervention and control group, we first have to provide evidence for measurement invariance.

Measurement invariance analyses can also refer to different points of time. This is essential for longitudinal analyses because researchers should ensure that their measurement instruments are equivalent over time. Changes in PHQ scores over different points of time can only be meaningfully interpreted if measurement invariance can be assumed. However, evidence of measurement invariance over time is scarce. For example, Downey, Hayduk [35] have examined longitudinal measurement invariance of the PHQ for family members of patients in intensive care units. They were unable to show invariance for either the PHQ-9 or the PHQ-8 and concluded that the questionnaire might not be adequate for the assessment of depression in this specific population. However, the authors only examined the fit of a constrained model without comparison to an unconstrained baseline model. A step-wise approach could be more adequate to analyze measurement invariance.

The aims of the current study were 1) to compare a one-factor structure to a two-factor structure for the PHQ-8 at one point of time (baseline assessment), 2) to provide evidence for measurement invariance across five points of time, including a baseline assessment and 2, 4, 6, and 12 month follow-up assessments separately for participants in the two study groups, and 3) to provide evidence for longitudinal measurement invariance between the intervention group and the control group.

## Methods

### Sample

Participants were recruited from 56 hospital wards and 39 general medical practices in two cities of Northern Germany (Greifswald and Luebeck) and one city in South Germany (Tuebingen). Research protocols were approved by the institutional review boards of all participating sites. From January 2017 to March 2018, study assistants proactively approached consecutive patients aged 18 to 64 years for an anonymous computerized health screening. Out of all eligible patients, a total of 13,763 (86.5%) patients started the screening and 12,828 participants completed it (detailed information on the screening has been published in [36]). The present analyses include all patients who 1) were eligible for one of two randomized brief intervention trials concerning harmful alcohol consumption and depressive symptoms (for more details, see [36, 37]), 2) gave their written informed consent to participate in the respective study, and 3) completed the baseline interview (*n* = 588). Of these, 46.6% (*n* = 274) reported more than 10 years of school education, 32.1% (*n* = 189) graduated after 10 years of schooling, 15.0% (*n* = 88) reported graduation after less than 10 years of schooling, 2.7% (*n* = 16) did not graduate from school, and 3.6% (*n* = 21) were not classifiable in regards to their level of schooling. Regarding their occupational status, 42.4% (*n* = 249) of the participants were fulltime employed, 15.1% (*n* = 89) reported part-time employment, 10.4% (*n* = 61) were unemployed, and 32.1% (*n* = 189) did not work (i.e. homemaker, retiree, student, or similar). After completing the baseline interview, participants were randomly assigned to either the intervention group (*n* = 291) or the control group (*n* = 297). The 2- and 4-month assessments were only conducted with the intervention group and were completed by 246 and 225 participants, respectively. The 6-month assessment was completed by 437 participants (intervention group: *n* = 215, control group: *n* = 222) and 447 participants completed the 12-month assessment (intervention group: *n* = 220, control group: *n* = 227). The sample characteristics are displayed in Table 1.

**Table 1:** Sample Characteristics

	Total sample				Intervention group				Control group			
	N	Sex (f)	Mean age (SD)	PHQ-8	N	Sex (f)	Mean age (SD)	PHQ-8	N	Sex (f)	Mean age (SD)	PHQ-8
Baseline	588	61.7%	39.3 (14.0)	13.9 (3.8)	291	65.6%	39.7 (14.0)	13.9 (3.8)	297	57.9%	38.9 (14.1)	13.8 (3.8)
2-months	246	65.0%	40.2 (13.9)	9.9 (5.0)	246	65.0%	40.2 (13.9)	9.9 (5.0)	–	–	–	–
4-months	225	62.7%	40.6 (13.8)	9.9 (5.1)	225	62.7%	40.6 (13.8)	9.9 (5.1)	–	–	–	–
6-months	437	60.2%	40.4 (14.0)	10.4 (5.2)	215	62.8%	40.4 (13.9)	10.4 (5.3)	222	57.7%	40.3 (14.1)	10.5 (5.1)
12-months	447	59.7%	40.4 (13.9)	10.2 (5.4)	220	62.7%	40.3 (13.8)	10.7 (5.5)	227	56.8%	40.5 (13.9)	9.8 (5.3)

Note. PHQ-8: sum score of the Patient-Healthcare Questionnaire-8 (standard deviation)

### Procedure

Participants were recruited by study nurses in general practices and general hospitals. They were asked to participate in a screening of multiple health risk behaviors, which was conducted as self-administered questionnaires via tablet computer. The assessment included socio-demographics, alcohol consumption, tobacco consumption, depressive symptoms, fruit and vegetable intake, and physical activity (see Supplementary Table 1, Additional file 1). Participants of this screening were asked for further participation in our studies if they reported depressive symptoms and alcohol consumption below a sum score of 20 of the Alcohol Use Disorder Identification Test [38].

Screening participants who had given written consent for further participation in the study were contacted during the following 2 weeks for another phone interview. After having completed the interview, participants were automatically and randomly assigned to the intervention and the control group. The intervention group was contacted again after 2 and 4 months after the baseline interview. At each point of time, another telephone interview was conducted and brief-intervention messages, tailored according to the participants’ responses, were sent via mail as well as email or message via short messenger service over the course of 4 months. During this time, participants of the control group were not contacted. Six months after the baseline interview, participants from both intervention and control group were contacted again via phone for the first follow-up assessment. The next follow-up assessment followed 12 months after the baseline interview. An overview of all instruments used during each telephone interview as well as the baseline interview will be included in the supplement, see Supplementary Table 1, Additional file 1.

### Measures

Depressive symptoms were assessed with the PHQ-8 (see Table 2 [19];). The 8 items refer to the diagnostic criteria of depressive disorders from the DSM-5 [14, 39], assessing depressed mood, anhedonia, significant change in weight or appetite, insomnia or hypersomnia, psychomotor agitation or retardation, fatigue or loss of energy, feeling of worthlessness or guilt, and diminished ability to think or concentrate (Table 2). Each response was rated on a 4-point Likert scale, assigning 0 to 3 points to each category (0 = “not at all”, 1 = “several days”, 2 = “more than half of the days”, 3 = “nearly every day”). The total sum score ranges from 0 to 24 points. Based on previous validation studies by Kroenke and Spitzer [8], a total score of ≥10 was chosen as an inclusion criterion for our studies. This cut-off has shown high sensitivity (≥ 99%) and specificity (91–92%) for diagnosing major depression in a large sample of primary care patients [8]. Previous validation studies further found excellent internal consistency with Cronbach’s α between .86 and .89 [7, 13, 40]. For the baseline assessment, the PHQ-8 was presented as a self-administered questionnaire on a tablet PC and participants were asked about a two-week episode of depressive symptoms in the past 12 months. The following assessments were conducted as computer-assisted telephone interviews and participants were asked about a two-week episode of depressive symptoms in the last 2 months in the 2-months and 4-months assessments, in accordance with the time frame of the intervention. For the 6-months and 12-months assessment, participants were again asked about a two-week episode of their depressive symptoms in the last 6 months.

**Table 2:** Patient Health Questionnaire eight-item depression measure (PHQ-8)

Over the last two weeks, how often have you been bothered by the following problems?					
		Not at all	Several days	More than half the days	Nearly every day
1	Little interest or pleasure in doing things	0	1	2	3
2	Feeling down, depressed, or hopeless	0	1	2	3
3	Trouble falling or staying asleep, or sleeping too much	0	1	2	3
4	Feeling tired or having little energy	0	1	2	3
5	Poor appetite or overeating	0	1	2	3
6	Feeling bad about yourself – or that you are a failure or have let yourself or your family down	0	1	2	3
7	Trouble concentrating on things, such as reading the newspaper or watching television	0	1	2	3
8	Moving or speaking so slowly that other people could have noticed, or the opposite – being so fidgety or restless that you have been moving around a lot more than usual	0	1	2	3

### Data analysis

Data management was performed with Stata version 14.1 [41]. Mplus version 7.31 [42] was used for the confirmatory factor analyses and measurement invariance analyses.

*Factor structure:* First, we used confirmatory factor analysis to examine the factor structure of the PHQ-8 by comparing a one-factor model to a two-factor model with a somatic factor (items 3, 4, 5, 7, and 8) and a non-somatic factor (items 1, 2, and 6). The Weighted Least Squares Mean and Variance adjusted (WLSMV) estimator was used instead of a maximum likelihood estimator because it is more suitable for categorical and highly skewed variables [43]. The Comparative Fit Index (CFI) ≥ .95 and the Root Mean Square Error of Approximation (RMSEA) ≤ .06 were used as indicators of good model fit [44, 45]. Because direct comparison of CFI and RMSEA between models estimated with WLSMV is not advisable, χ^2^ difference testing was used for model comparisons, by using the DIFFTEST option of Mplus.

**Fig. 1:**
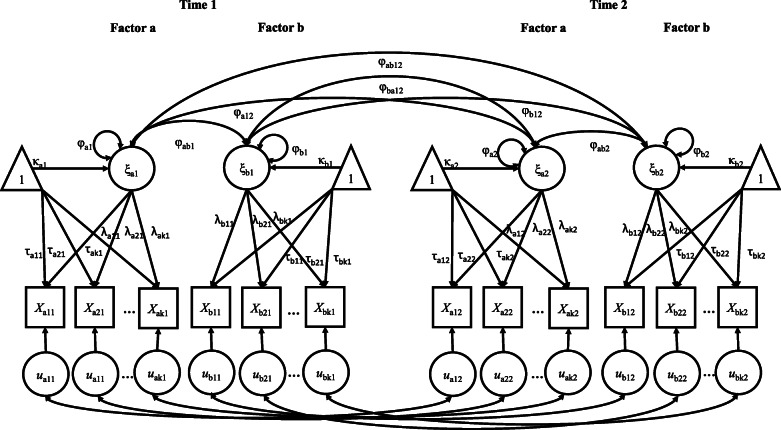
Exemplary longitudinal confirmatory factor analysis model with two points of time. Note. 1 = threshold/mean score on each item, ξ = the latent common factor, X = the observed indicators, k = number of observed indicators, u = the item residuals, λ = factor loadings, τ = the item thresholds, φ = factor correlations

*Measurement invariance across time:* Longitudinal measurement invariance analyses were performed in a framework of structural equation modeling (see Fig. 1). First, measurement invariance was analyzed across time separately for the intervention group and the control group. Invariance testing is a sequential procedure of increasingly constraining measurement parameters (factor loadings, item thresholds, and residual variances) to be equal across groups with each level of invariance. In the case of longitudinal invariance testing, the parameters are constrained to equality across time. Different levels of invariance are assessed by consecutive comparisons of measurement models from the least to the most restrictive model.

In a first step, *configural* invariance was examined by fixing the factor structure for each measurement point to equality but freely estimating the model parameters. Since the PHQ-8 does not contain continuous variables, the procedure for the following steps of the invariance analysis were adapted according to Millsap [46]. Thus, in a second step, *metric* invariance was analyzed by constraining the factor loadings to equality across time, fixing residual variances at one in one group while freeing them in the other groups, and fixing factor means at zero in one group while freeing them in the other groups. In this analysis, groups refer to the five measurement points. Furthermore, the first threshold of each item and the second threshold of the item used to set the metric of the factor were held equal across time. To establish *scalar* invariance, factor loadings and thresholds were fixed to equality across time. Again, the factor means were fixed to zero and residual variances were fixed to one in one group but were freely estimated in the other groups. In a last step, additionally to the previous restraints, the residual variances were fixed to one in all groups to examine the model for *strict* invariance. χ^2^ difference testing was used as the indicator of deteriorations of model fit with increasingly restricted models. A non-significant result on the difference testing would indicate that the models do not differ substantially and thus, full measurement invariance can be assumed for the model. However, significant results on the χ^2^ difference test do not necessarily mean that the assumption of measurement invariance needs to be fully rejected. Instead, it is often possible to examine the model for partial invariance [47]. Often partial invariance can be shown by freeing individual parameters from the consecutive constraints.

*Measurement invariance across time and between groups:* The longitudinal models were then applied to a series of Multiple Group Confirmatory Factor Analyses (MGCFA) to analyze measurement invariance simultaneously across time and between the intervention and the control group. For this analysis, the study group (i.e. assignment to intervention and control group) was included as the grouping variable. Because the 2- and the 4-month assessments were only conducted with the intervention group, these measurement points could not be included in these analyses. Therefore, three points of time were used in this examination of measurement invariance across time and between groups, namely the baseline assessment, the 6-month follow-up and the 12-month follow-up.

## Results

### Factor structure

First, we used CFA to compare a one-factor measurement model with a two-factor measurement model for the baseline assessment (Table 3). Both models showed a good fit to the data, one-factor model: χ^2^(650) = 999.00, *p* < .001, CFI = .957, RMSEA = .030; two-factor model: χ^2^(615) = 870.54, *p* < .001, CFI = .969, RMSEA = .027. As stated above, model fit indices are not adequate tools for model comparison with the WLSMV estimator and a χ^2^ difference test should be used instead. The results of the difference testing indicate a significantly better fit for the two-factor model, Δχ^2^(35) = 137.77, *p* < .001. Therefore, the following analyses were conducted using the two-factor model.

**Table 3:** Model Comparison for One- vs Two-Factor Measurement Model

Model	χ^2^(df)	CFI	RMSEA	Δχ^2^(df)	*p*	*N*
1) one factor	165.61 (20)	.989	.111			
2) two factor	129.54 (19)	.992	.099	95.05 (1)	< .001	588

Note. *CFI* Comparative Fit Index, *RMSEA* Root Mean Square Error of Approximation

### Measurement invariance across time

First, we tested longitudinal measurement invariance in separate analyses for the intervention group and the control group (Tables 4 and 5). The configural model showed a good fit to the data for both groups, intervention group: χ^2^(615) = 794.54, *p* < .001, CFI = .969, RMSEA = .032; control group: χ^2^(213) = 321.06, *p* < .001, CFI = .958, RMSEA = .041. After restricting the factor loadings to equality to examine the models for metric invariance, the fit indices still indicated a good fit to the data, intervention group: χ^2^(647) = 835.68, *p* < .001, CFI = .967, RMSEA = .032; control group: χ^2^(227) = 344.08, *p* < .001, CFI = .955, RMSEA = .042. However, the χ^2^ difference test was significant for both groups, indicating a substantial change compared to the configural model, intervention group: Δχ^2^(32) = 50.18, *p* = .02; control group: Δχ^2^(14) = 27.98, *p* = .01. Thus, we could not assume full metric invariance across time for the PHQ-8 and decided to test for partial invariance instead.

**Table 4:** Invariance Testing Across Time for the Two-Factor Model of the PHQ-8 in the Intervention Group

Model	χ^2^(df)	CFI	RMSEA	Model comparison	Δχ^2^(df)	*p*
All assessments						
3) configural	794.54 (615)	.969	.032			
4) metric	835.68 (647)	.967	.032	3 vs. 4	50.18 (32)	.02
Without baseline						
5) configural	509. 5 (388)	.977	.035			
6) metric	528.3 (406)	.977	.034	5 vs. 6	19.47 (18)	.36
7) scalar	566.82 (448)	.977	.032	6 vs. 7	41.2 (42)	.51
8) strict	599.43 (478)	.977	.031	7 vs. 8	42.82 (30)	.06

Note. *CFI* Comparative Fit Index, *RMSEA* Root Mean Square Error of Approximation

**Table 5:** Invariance Testing Across Time for the Two-Factor Model of the PHQ-8 in the Control Group

Model	χ^2^(df)	CFI	RMSEA	Model comparison	Δχ^2^(df)	*p*
All assessments						
9) configural	321.06 (213)	.958	.041			
10) metric	344.08 (227)	.955	.042	9 vs. 10	27.98 (14)	.01
Without baseline						
11) configural	172.10 (90)	.963	.062			
12) metric	175.39 (96)	.964	.059	11 vs. 12	3.71 (6)	.72
13) scalar	182.55 (110)	.967	.053	12 vs. 13	10.53 (14)	.72
14) strict	178.74 (120)	.973	.045	13 vs. 14	9.06 (10)	.53

Note. *CFI* Comparative Fit Index, *RMSEA* Root Mean Square Error of Approximation

This would usually be achieved by freeing the equality restrictions of individual parameters. The modification indices suggested freeing equality restriction for the baseline assessment. Unfortunately, the modification indices were substantially higher for all factor loadings of the baseline assessment compared to the other time points and we were unable to distinguish individual items that may be responsible for the non-invariance. We had to free all factor loadings of the baseline assessment in order to have a non-significant χ^2^ difference test. This did not justify to assume partial metric invariance across time for the PHQ-8. However, we repeated the invariance analysis without the baseline assessment, i.e., only including the 2-month, 4-month, 6-month, and 12-month assessments for the intervention group (see Table 4) and the 6-month and 12-month assessments for the control group (see Table 5). For this analysis, strict invariance was shown for both groups; intervention group: χ^2^(478) = 599.43, *p* < .001, CFI = .977, RMSEA = .031, Δχ^2^(30) = 42.82, *p* = .06; control group: χ^2^(120) = 178.74, *p* < .001, CFI = .973, RMSEA = .045, Δχ^2^(10) = 9.06, *p* = .53.

### Measurement invariance across time and between groups

Next, we tested for invariance both across time and between groups (see Table 6). Because the experimental group was introduced as the grouping variable (i.e. intervention vs. control group) but the 2-month and 4-month follow-up interview was only conducted with the intervention group, the following analyses were restricted to the 6-month and 12-month assessments.

**Table 6:** Invariance Testing for the Two-Factor Model of the PHQ-8 Across 6 & 12 Months and Between Groups

Model	χ^2^(df)	CFI	RMSEA	Model comparison	Δχ^2^(df)	*p*
15) configural	291.79 (180)	.976	.051			
16) metric	316.04 (214)	.979	.045	15 vs. 16	36.95 (34)	.33
17) scalar	353.03 (256)	.980	.040	16 vs. 17	47.95 (42)	.24
18) strict	370.13 (272)	.979	.039	17 vs. 18	24.35 (16)	.08

Note. *CFI* Comparative Fit Index, *RMSEA* Root Mean Square Error of Approximation

The MGCFA across the 6-month and 12-month assessments revealed strict invariance between the intervention and control group, χ^2^(272) = 370.13, *p* < .001, CFI = .979, RMSEA = .039; Δχ^2^(16) = 24.35, *p* = .08. Due to the lack of longitudinal invariance with the inclusion of the baseline assessment, the measurement invariance between groups for the baseline assessment was examined with a separate analysis (Table 7). This analysis showed scalar invariance between groups, χ^2^(59) = 108.12, *p* < .001, CFI = .995, RMSEA = .053; Δχ^2^(13) = 19.48, *p* = .11. However, the χ^2^ difference test for the strict invariance model was significant (Δχ^2^(8) = 27.24, *p* < .001), thus the assumption of strict invariance for the baseline assessment had to be rejected. Overall, we can conclude that we found measurement invariance between intervention and control group at baseline and, analyzed separately, across the 6-month and 12-month follow-up.

**Table 7:** Invariance Testing for the Two-Factor Model of the PHQ-8 Across 6 & 12 Months and Between Groups

Model	χ^2^(df)	CFI	RMSEA	Model comparison	Δχ^2^(df)	*p*
19) configural	99.01 (38)	.994	.074			
20) metric	97.80 (46)	.995	.062	19 vs. 20	7.14 (8)	.52
21) scalar	108.12 (59)	.995	.053	20 vs. 21	19.48 (13)	.11
22) strict	119.88 (67)	.995	.052	21 vs. 22	27.24 (8)	< .001

Note. *CFI* Comparative Fit Index, *RMSEA* Root Mean Square Error of Approximation

## Discussion

A two-factor structure with a somatic and a non-somatic factor showed the best model fit for all measurement models in our analyses. Full measurement invariance was only achieved across the 2-, 4-, 6-, and 12-month assessments. Including the baseline assessment into the model resulted in a substantial deterioration of the model fit at the metric invariance level. Thus, only the same factor structure could be assumed across all assessments.

So far, studies on the measurement invariance of the PHQ-9 have consistently shown invariance across sociodemographic variables [29]. Although the number of studies is still small, this suggests that PHQ-9 scores can be meaningfully compared across sociodemographic groups. However, far less is known about the longitudinal measurement invariance of both PHQ-8 and PHQ-9. For example, Downey, Hayduk [35] reported non-invariance of one-factor models for both questionnaires while Schuler, Strohmayer [48] found at least partial scalar invariance for a one-factor model of the PHQ-9. Gonzalez-Blanch, Medrano [49] even found strict invariance for a one-factor model of the PHQ-9 between two assessments. These differences might be due to different methodological approaches (i.e. one-step or four-step analysis) or differences in the sample populations (e.g. clinical or non-clinical populations). Our results demonstrate that longitudinal invariance can also be established for a two-factor model of the PHQ-8 (for four of five assessments) and further include measurement invariance between experimental groups which is crucial to show that differences between intervention and control group reflect the inferred underlying construct.

The reported lack of invariance across the baseline assessment and all other assessments could have several explanations, one of which being the different modes of presentation of the PHQ-8 (self-administered questionnaire versus telephone interview). Effects of presentation modes have been investigated for several tests and questionnaires. For the PHQ-9, there is evidence that the telephone version is comparable to a paper-pencil version of the questionnaire [18]. However, to our knowledge, no such examination has been conducted for the PHQ-8 so far. Furthermore, no data exists on how a computerized assessment may differ from telephone assessments. This could have important implications for the PHQ. Future research could examine if different modes of presentation require different cut-off points for screening depressive symptoms with the PHQ.

It is possible, that the different timeframes for the items (i.e., the past 12 months at baseline, the past 2 months for the 2- and 4-months assessments, and the past 6 months for the 6- and 12-months assessments) contributed to the lack of longitudinal invariance. Our results could suggest that the retrospective assessment of depressive symptoms could be biased for longer periods of time such as the 12-months interval. This seems reasonable considering that an accurate recall of symptoms becomes increasingly difficult over longer periods. Possibly, our results did not show longitudinal invariance with the baseline assessment because participants were only asked to think about such a long timeframe at the baseline assessment. However, the strict invariance across the follow-up assessments suggests that smaller differences in the time frames for the PHQ-8 might not be a problem for longitudinal analyses.

Finally, completing the initial screening and agreeing to participate in a study focusing on depressive symptoms could have resulted in a heightened self-awareness of participants regarding their mental health. This might have led to participants having different perceptions of the respective items about depression in contrast to the initial assessment at which the majority of participants may not have thought about depressive symptoms before. It is important to note that measurement invariance was shown between the intervention and the control group. Therefore, it is highly unlikely that the application of questions about health behaviors caused biased responses only in the intervention group [50]. Although the control group did not receive the intervention, the mere participation in the study and the baseline assessment were sufficient to change participants’ self-awareness about their own mental health states at the follow-up assessments, for participants in the intervention as well as the control group. This result shows that group comparisons between intervention and control group at 6-month, and 12-month follow-up assessments and, for separate analyses, at the baseline assessment for the PHQ-8 mean score are explicitly meaningful [51].

## Conclusions

The configural invariance for all five points of time shows that the PHQ-8 reliably captures the same conceptual framework (i.e., the same factor structure) when measured over time. However, the lack of metric invariance (i.e., factor loadings can not be assumed to be equal across time) means that the associations and patterns mapping the items and factors can not be assumed to be equal across the baseline and the follow-up assessments. Furthermore, we can not conclude that the PHQ-8 has the same operational definition across time due to the lack of scalar invariance (i.e., item thresholds can not be assumed to be equal across time). Nevertheless, we were able to establish strict longitudinal invariance across the 2-, 4-, 6- and 12-month assessments and between groups across the 6- and 12-month assessments. This emphasizes the influence of the varying factors between the baseline and the follow-up assessments on our results, such as the different modes of presentation (self-administered vs. telephone interview). Rather than the longitudinal design, it is very likely that the lack of invariance was caused by these factors. Altogether, the results indicate that the PHQ can be compared across time and between groups – at least when it is used under similar conditions (presentation mode, timeframe of the items, assessment setting). However, researchers interested in longitudinal measurements of the PHQ-8 should be careful with varying conditions between measurement points. Future research should investigate the validity and possible differences of a self-administered paper-pencil version, the digital version, and the telephone interview of the PHQ-8.

## Supplementary Information

**Additional file 1: Supplementary Table 1.** List of instruments for all assessments in both studies across all measurement times.

## Data Availability

The datasets used and/or analyzed during the current study are available from the corresponding author on reasonable request.

## Abbreviations

PHQ: Patient Health Questionnaire
CFA: Confirmatory Factor Analysis
MGCFA: Multiple Group Confirmatory Factor Analysis
WLSMV: Weighted Least Squares Mean and Variance adjusted estimator
CFI: Comparative Fit Index
RMSEA: Root Mean Square Error of Approximation
AUDIT: Alcohol Use Disorders Identification Test
