# Supplementary material for: Longitudinal measurement invariance of the patient health questionnaire in a German sample
Source: BMC Psychiatry. 2021 Aug 4;21:386. doi: 10.1186/s12888-021-03390-0 (PMC8335884; doi:10.1186/s12888-021-03390-0)
Supplement: Supplementary file 1 — Additional file 1: Supplementary Table 1. List of instruments for all assessments in both studies across all measurement times. [file 12888_2021_3390_MOESM1_ESM.docx]

**Supplementary Table 1**

*Instruments for all assessments in both studies across all measurement times*

| Instruments | Baseline | 2- & 4-month | 6-month | 12-month |
| --- | --- | --- | --- | --- |
| Depression |  |  |  |  |
| CIDI screening items  Wittchen et al. 1995 ^1^ | x^a^ | x | x | x |
| PHQ-8  Kroenke & Spitzer 2002; Ger. version: Löwe et al. 2002 | x^a^ | x | x | x^c^ |
| Stage of willingness to change for depression preventive behaviors  Self-designed; based on Levesque et al. 2011 | x^b^ | x | x | x |
| Perceived self-efficacy  Self-designed | x^b^ | x | x | x |
| Perceived outcome expectations  Self-designed | x^b^ | x | x | x |
| Processes of change for depression prevention  Self-designed | x^b^ | x |  |  |
| Self-stigma of depression  (Self-stigma of mental illness scale; SSMI-SF)  Corrigan et al. 2012; adapted Ger. version ^2^ | x^b^ |  | x | x |
| Loneliness (UCLA Loneliness Scale)  Russell et al. 1978; rev. Peplau & Cutrona 1980; Ger. version Döring & Bortz 1993 | x^b^ |  | x | x |
| Positive and negative affect (PANAS)  Watson et al. 1988; adapted Ger. version Krohne et al. 1996 | x^b^ |  | x | x |
| Wellbeing  (WHO-5 wellbeing index)  Bech 2004; Ger. version: Brähler et al. 2007 ^3^ | x^b^ |  | x | x |
| CIDI Section E  Wittchen et al. 1995 |  |  |  | x |
| Alcohol |  |  |  |  |
| AUDIT ^4^  Saunders et al 1993; Ger. version: Rumpf et al. 2010  AUDIT-C ^4^  Bush et al. 1998 | x^a^ | x^ITE^ | x^ITE^  x^ACT^ | x^ITE^  x^ACT^ |
| Stage of willingness to change  DiClemente et al. 1991; adapted Ger. version: Freyer-Adam et al. 2016 | x^b, ITE^ | x^ITE^ | x^ITE^ | x^ITE^ |
| Perceived self-efficacy  (Alcohol-Abstinence-Self-Efficacy-Scale; AASE)  DiClemente et al. 1994; Ger. version: Bott et al. 2003, short-scale by Baumann et al. 2013 | x^b, ITE^ | x^ITE^ | x^ITE^ | x^ITE^ |
| Decisional balance  (Alcohol Decisional Balance Scale; ADBS-G)  King & DiClemente 1993; Ger. version: Hannöver et al. 2003, gekürzte Version nach Baumann et al. 2013 | x^b, ITE^ | x^ITE^ | x^ITE^ | x^ITE^ |
| Stages of change for alcohol consumption  DiClemente et al. 1996; Ger. Short-scale Freyer et al. 2006 | x^b, ITE^ | x^ITE^ |  |  |
| Self-stigma of alcohol dependence  (Self-stigma in alcohol dependence; SSAD-SF)  Corrigan et al. 2012; adapted Ger. version Schomerus et al. 2011 | x^b, ITE^ |  | x^ITE^ | x^ITE^ |
| Other |  |  |  |  |
| Socio-demographics (age, gender, family status, education, employment)  Based on the standards of socio-demographics 2010 | x^a^ |  |  |  |
| Height and weight  Self-designed | x^a^ |  |  |  |
| Importance of personal health  Henrich & Herschbach 2000 | x^a^ |  |  |  |
| General health  (1^rst^ item Short Form 36)  McHorney et al. 1993; Ger. version: Bullinger 1998 | x^a^ |  |  |  |
| Tobacco consumption ^6^, Number of cigarettes per day ^6^, Onset  Medical consultation  Self-designed  FTND (1^st^ item) ^6^  Heatherton et al. 1991; Ger. version: Schumann et al. 2010 | x^a^  x^a^  x^a^ |  | x  x | x  x |
| Fruit & vegetable consumption ^8^  WHO (o.J.); self translated | x^a^ |  | x | x |
| Physical activity ^7^  (Godin Leisure-Time Exercise Questionaire)  Godin 2011; self translated | x^a^ |  | x | x |
| Use of Internet & mobile phone  Self-designed | x^a^ |  |  |  |
| Intake of medication  Self-designed | x^b, ITE^ |  | x | x |
| Asking for help  Self-designed | x^b, ITE^ |  | x | x |
| Health-related quality of life  (Veterans RAND 12 health questionnaire; VR-12)  Kazis et al. 2004; Ger. version: Buchholz & Kohlmann 2015 ^5^ | x^b^ |  | x | x |
| Limitations due to psychological, physical, substance-related problems  (M-CIDI Section Q, final questions) Wittchen et al. 1995 | x^b^ |  | x | x |
| Inability to work during the last 6 months  Self-designed | x^b^ |  | x | x |
| Subjective social Status  Adler et al. 2000; Ger. version: Hoebel et al. 2015 | x^b^ |  | x | x |
| Preferred mode of contact (SMS/ e-mail) | x^b^ |  |  |  |

*Note*. ^ITE^ only for participants of study 1; ^ACT^ only for participants of study 2;^a^ Baseline screening before study inclusion in general practices and hospitals with self-administered survey via tablet computer; ^b^ Baseline assessment after study inclusion via phone, ^c^ PHQ regarding the last 6 months and additionally for the last 2 weeks if the only/the worst phase did not occur during the last 2 weeks; deviations from the original instrument: ^1^ the 3^rd^ item was changed from “I think” to “this means”; ^2^ German version of Schomerus; “psychologic disease” was replaced by “depression”; ^3^ Scale of 1-6; ^4^ additional continuous response categories for items 1 to 3 and gender-specific inquiry for item 3; ^5^ phone interview derived from the paper-pencil version. Additionally, some adaptations for wording were necessary. Multiple consultations with the authors oft he German versions ensued; ^6^ Addition for the hospital setting: „Please respond tot he question in regards tot he last 4 weeks before your inpatient treatment”; ^7^ Additional information about the typical duration of the activity in minutes; Introductory explanation of different kinds of activity based on relative and absolute intensity (CDC); Adaptation of examples of activities based on Ainsworth et al. (2011). Compendium of Physical Activities: an update of activity codes and MET intensities. Medicine and Science in Sports and Exercise, 32, 498-516; ^8^ Additional explanation of one serving size
